# Optimizing shelf life conditions for anthocyanin-rich tomatoes

**DOI:** 10.1371/journal.pone.0205650

**Published:** 2018-10-11

**Authors:** Tina Petric, Claudia Kiferle, Pierdomenico Perata, Silvia Gonzali

**Affiliations:** PlantLab, Institute of Life Sciences, Scuola Superiore Sant’Anna, Pisa, Italy; Universidade do Minho, PORTUGAL

## Abstract

Shelf life is the time a product can be stored without losing its qualitative characteristics. It represents one of the most critical quality traits for food products, particularly for fleshy fruits, including tomatoes. Tomatoes’ shelf life is usually shortened due to fast over-ripening caused by several different factors, among which changes in temperature, respiration and pathogen exposure. Although tomatoes usually do not contain anthocyanins, varieties enriched in these antioxidant compounds have been recently developed. The anthocyanin-rich tomatoes have been shown to possess a significantly extended shelf life by delayed over-ripening and reduction of the susceptibility to certain pathogens. In the present work, we compared different conditions of postharvest storage of anthocyanin-rich tomato fruits with the aim to understand if the added value represented by the presence of the anthocyanins in the fruit peel can be affected in postharvest. For this purpose we used an anthocyanin-enriched tomato line derived from conventional breeding and took into consideration different light and temperature conditions, known to affect fruit physiology during postharvest as well as anthocyanin production. Several quality traits related to the fruit ripening were measured, including anthocyanin and carotenoid content, pH, titratable acidity and total soluble solids. In this way we identified that the most suitable fruit storage and postharvest anthocyanin accumulation were obtained through exposure to cool temperature (12° C), particularly in the presence of light. Under these parameters, tomato fruits showed increased anthocyanin content and unchanged flavour-related features up to three weeks after harvesting.

## Introduction

Tomato (*Solanum lycopersicum* L.) is the second most cultivated and one of the most consumed vegetables worldwide [1]. It also represents an important model for fleshy fruit ripening [2]. Shelf life is the time between production and consumption of a product during which it can be stored without losing its satisfactory quality and safeness. It represents one of the most critical quality traits for fleshy fruits, and can be affected by different factors such as exposure to unsuitable temperature and humidity or to pathogens, which can promote over-ripening. Fast over-ripening leads to reduced shelf life and therefore represents a relevant challenge for the tomato industry [3, 4].

One of the most used approaches to extend tomato shelf life is based on harvesting fruit at the mature green stage followed up by low-temperature storage and, eventually, ethylene exposure to induce ripening [4]. Transgenic approaches, such as those exploiting mutations in genes involed in ripening, like *rin* (*ripening inhibitor*), *nor* (*non-ripening*), *Cnr* (*Colorless non-ripening*), *Nr* (*Never-ripe*), *Gr* (*Green-ripe*) and *alc* (*alcobaca*), are also utilized to slow-down ripening and thus extend shelf life [3, 4]. Unfortunately, all these approaches result in lower levels of fruit pigments and sugars and, consequently, loss of flavour, making fruit less appealing to the consumer.

Tomato is a climacteric fruit with oxidative processes taking place during ripening. Changes in the oxidative metabolism with the accumulation of reactive oxygen species (ROS) are often observed during ripening [5]. ROS are natural byproducts of oxygen metabolism, with important roles in cell signalling and homeostasis. However, over-accumulation of ROS can lead to membrane deterioration, lipid peroxidation and, eventually, cell death [6]. To maintain homeostasis, plants have developed complex systems to control ROS production or to scavenge them. It has been shown that flavonoids have high ROS-scavenging ability thanks to their hydroxyl groups which can react with the most damaging radical species. Hence, this property can be exploited to counteract ROS production during ripening and thus to extend postharvest shelf life [4].

Anthocyanins are water-soluble flavonoids which give red, purple and blue pigmentation to several different fruits and vegetables. They are powerful antioxidants with high nutraceutical value due to their anti-inflammatory and anti-carcinogenic effects on human health [7, 8]. Unfortunately, tomato only contains negligible amounts of anthocyanins in its fruits [1, 9]. However, some progress has been made to increase anthocyanin content in tomatoes, either exploiting genetic engineering or conventional breeding, with encouraging results [9–11]. The best-known biotechnological approach is the fruit-specific expression of two anthocyanin synthesis related transcription factors (TFs), *Delila* (*Del*) and *Rosea1* (*Ros1*), from snapdragon, leading to purple-colored fruits [12]. Since consumers are often reluctant to accept genetically engineered plants, efforts to enrich tomato fruits with anthocyanins by conventional breeding were also undertaken [9, 10]. New varieties were produced by exploiting the ability of some wild tomato species to produce higher amounts of anthocyanins, especially in the fruit [13]. In particular, the dominant gene *Aft (Anthocyanin fruit)*, derived from *Solanum chilense* [14, 15], which confers anthocyanin accumulation in the fruit peel, and the recessive gene *atv (atroviolacea)* from *Solanum cheesmaniae* (L. Riley) Fosberg [16], which enhances anthocyanin accumulation in vegetative tissues, were introgressed through interspecific crosses in *S*. *lycopersicum*. Whereas the *Aft* gene likely encodes an R2R3 MYB activator of the anthocyanin biosynthetic pathway, whose precise identity is still under debate [17, 18], the *atv* gene has been recently identified and encodes for an R3 MYB repressor of the pathway [19, 20]. A further cross between the *Aft* and *atv* lines led to the double *Aft/Aft atv/atv* genotype (commercially known as “Indigo™ Rose” or “Sun Black™”) characterized by a highly pigmented fruit phenotype [13, 21–23]. In these high anthocyanin varieties, anthocyanin accumulation is restricted to fruit peel and represents a conditional phenotype, induced by high light and/or low temperatures [21]. Interestingly, these anthocyanin-rich tomatoes resulted to have a two-fold longer shelf life in comparison to wild-type fruits, thanks to increased resistance to necrotrophic pathogens and slower ripening [10, 11].

The regulation of the flavonoid biosynthesis is well understood in several plant species. Crucial players in this process are R2R3 MYB TFs which, in response to specific cellular or environmental signals, initiate the pathway by activating in a coordinate manner the expression of the structural biosynthetic genes [24]. Some of these TFs interact with bHLH and WD40 proteins forming multiprotein complexes (the so-called MBW complexes) responsible for the transcriptional regulation of specific structural genes such as the late anthocyanin biosynthetic genes (e.g. *flavonoid 3′5′-hydroxilase*, *F3′5′H*, *dihydroflavonol 4-reductase*, *DFR*, and *leucoanthocyanidin dioxygenase*, *ANS*) [25]. Recently, different R2R3 MYB TFs (e.g. SlANT1, SlAN2), bHLH factors (e.g. SlAN1 and SlJAF13) and WD40 proteins (e.g. SlAN11) have been identified as possible candidates for the MBW complex regulating anthocyanin synthesis in tomato [1, 26, 27].

Light and temperature represent two major environmental parameters able to affect anthocyanin synthesis. Light shows a promoting role either directly activating R2R3 MYB TFs [1, 28, 29] or preventing their degradation regulated under dark by the ubiquitin E3 ligase COP1 protein [30–31]. On the other hand, low temperatures can induce anthocyanin pigmentation activating the bHLH component of the MBW complex, such as in apple skin [32], whereas, on the contrary, high temperatures can slow down the process [33, 34]. Understanding whether and how light and temperature can specifically influence anthocyanin accumulation during tomato fruit ripening and storage can be therefore worth of studying. In the present work, we stored anthocyanin-rich *Aft/Aft atv/atv* tomatoes, harvested at the breaker stage, in different conditions of light and temperature, with the aim of establishing a protocol for optimal postharvest storage of these fruits. Our goal was, in particular, to find conditions that do not affect in a negative way, rather possibly improve, the anthocyanin content during shelf life without threatening the other quality traits of the fruits.

## Materials and methods

### Plant material, growth conditions and fruit storage

Plants of *S*. *lycopersicum Aft/Aft atv/atv* producing anthocyanin-rich fruits were used. Tomato plants were hydroponically grown in a glasshouse located in Pisa (Italy) (latitude 43°43'N; longitude 10°23'E) during the summer/autumn seasons of 2017. The composition of the nutrient solution used for drip irrigation was as follows: macronutrients (concentrations in mM): 12.0 N-NO_3_, 1.0 P-H_2_PO_4_, 8.4 K, 5.0 Ca, 1.5 Mg, 3.8 S-SO_4_; micronutrients (concentrations in μM): 15.0 Fe, 20.0 B, 3.0 Cu, 10.0 Zn, 10.0 Mn, 1.0 Mo. Technical-grade inorganic salts were used. Electrical conductivity and pH of newly-prepared nutrient solution were respectively 0.246 S m^-1^ and 5.6.

Tomato fruits were randomly collected at the breaker stage, surface-sterilized by immersion in a 4% (v/v) solution of sodium hypochlorite for 10 min, rinsed in sterile distilled water and dried on blotting paper. Single fruits were then placed into 0.5 L transparent plastic boxes and stored in different environmental conditions: 12° C + light, 12° C + dark, room temperature (RT) + light, and RT + dark. For the conditions of 12° C, fruits were placed in an incubator, with 16 h/8 h photoperiod, 80 μmol m^-2^s^-1^ photon flux density and 70% relative humidity (RH). For the RT conditions, fruits were placed in a climate growth chamber at 24° C with 16 h/8 h photoperiod, 80 μmol m^-2^s^-1^ photon flux density and 55% to 60% RH. Although RH conditions were different between the incubator and the growth chamber, in both the cases fruits were stored inside plastic boxes where RH was close to saturation, as indicated by the presence of condensation on the box lids. Fruits in light condition were placed in non-covered transparent plastic boxes, while those in dark condition were placed in boxes covered with aluminium foil. At t0 and after one, two and three weeks of storage, fruits were visually inspected, photographed and weighted. Fruits incubated at 12° C were also characterized for the anthocyanin content, some biochemical traits and the expression of genes involved in the anthocyanin biosynthetic pathway and in abscisic acid (ABA) and ethylene synthesis. In detail, fruits were divided in two parts. Half of each fruit (peel and flesh) was homogenized in a blender, stored at -20° C and used for the biochemical determinations, whereas peel samples obtained from the remaining half of the fruits were frozen in liquid nitrogen and stored at -80° C until RNA extraction. All the biochemical analyses, excluding carotenoid determination, were performed on the fruit homogenate supernatant obtained after two consecutive centrifugations at 7,800 g for 10 min.

### Weight loss

Fruit fresh weight (FW) was measured at the time of collection (t0) and after one, two and three weeks of storage in the different conditions. Changes in FW were expressed as a percentage of weight reduction from FW measured at t0.

### Determination of anthocyanin content

Anthocyanin content in fruit aqueous extracts was assessed by measuring total monomeric anthocyanins using the pH-differential method [35] and expressed as mg L^-1^ of petunidin 3-(p-coumaroyl-rutinoside)-5-glucoside (extinction coefficient 17,000; molar mass 0.934 kg mol^-1^ [36]).

### Determination of carotenoid content

Carotenoid extraction was performed according to [37], with minor modifications. Briefly, homogenized fruit samples were weighted into glass tubes wrapped with aluminum foil to exclude light and a mixture of hexane/acetone/methanol (2:1:1 v/v/v) containing 0.05% butylated hydroxytoluene was added to solubilize the carotenoids (40:1 v/w liquid:solid ratio). Samples were shaken ON at 4° C, then distilled water (20% of volume) was added and the mixture was mixed 5 min at RT to allow phase separation. The absorbance of the non-polar layer containing carotenoids was measured at 520 nm versus a blank of hexane solvent. The total content of carotenoids was quantified according to the method reported by [38] (mean absorption coefficient 135,310; average molar mass 0.548 kg mol^-1^).

### Determination of pH, titratable acidity and total soluble solids

The total soluble solids (TSS) content was assessed in fruit aqueous extracts using a refractometer (RL3 type, PZO Warszawa, Poland) and expressed as %. The pH of the fruit samples was assessed using a digital pH meter (VWR International, Germany). Titratable acidity (TA) was measured according to AOAC method 942.15 [39] and expressed as % citric acid.

### Expression analysis by quantitative RT-PCR (qPCR)

Total RNA was extracted from 100 mg fruit peel samples using the “TRI Reagent” (Sigma-Aldrich Co., St. Louis, USA) and following the protocol suggested by the manufacturer. RNA integrity was checked by electrophoresis on a standard 1% agarose gel. RNA quantity and purity were assessed by measuring UV absorption at 260, 280 and 230 nm using a Multiscan Go (Thermo Scientific, Waltham, MA, USA). RNA was then subjected to DNAse treatment and reverse transcription using the “Maxima First Strand cDNA Synthesis Kit” (Thermo Fisher Scientific, Waltham, MA, USA). qPCR was performed with an ABI Prism 7300 Sequence Detection System (Applied Biosystems, Foster City, CA, USA) using the “PowerUp SYBR Green Master Mix” (Thermo Fisher Scientific) and the primers listed in S1 Table. *S*. *lycopersicum Elongation factor 1-alpha* (*SlEF1A*) was used as reference gene (S1 Table). The relative quantification of each individual gene expression was performed using the ΔΔCt method as described in the ABI PRISM 7700 Sequence Detection System User Bulletin #2 (Applied Biosystems).

### Statistical analysis

All the biochemical analyses were done in five biological replicates, whereas the qPCR analysis was carried out using three biological replicates. In both the cases, each biological replicate corresponded to a single fruit sample. Collected data were analyzed using analysis of variance (ANOVA) in the StatGraphics Centurion XVII program. Comparisons among the treatments with significant differences were done using Tukey honest significant difference (HSD) at p < 0.05 level.

## Results

### Fruit modifications during the three weeks of storage

**Fig 1:**
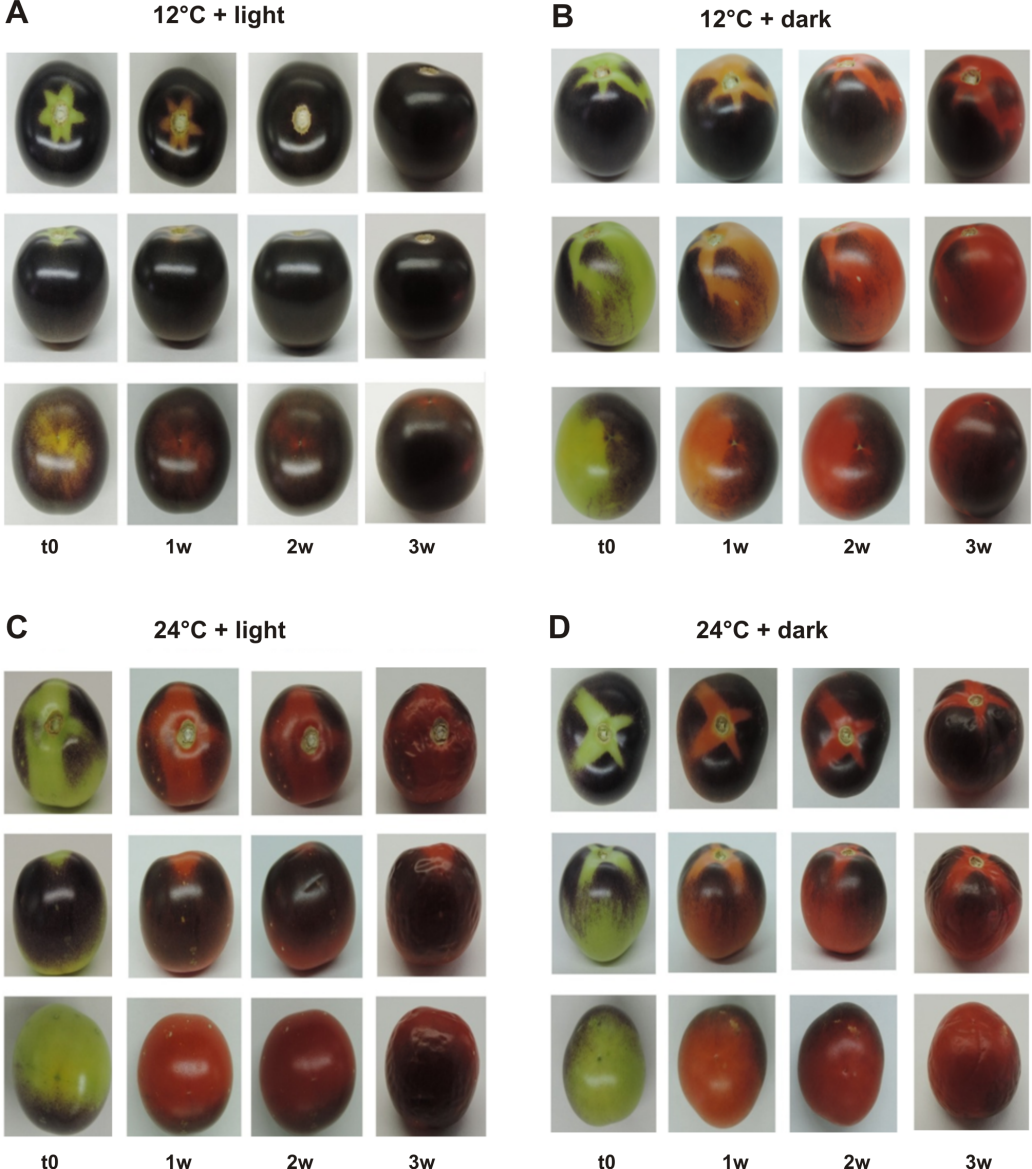
Fruit phenotype in the different storage conditions. *Aft/Aft atv/atv* fruits at the time of collection—t0 (breaker stage)—and after one, two and three weeks (w) of storage at 12° C, light (A) and dark (B), and at RT (corresponding to 24° C), light (C) and dark (D). For each condition, a representative fruit, photographed under different perspectives (top, lateral and bottom view), is shown.

*Aft/Aft atv/atv* fruits, collected at the breaker stage (t0), were stored for three weeks under four different conditions: 12° C in the light, 12° C in the dark, RT (corresponding to 24° C) in the light, RT in the dark, and visual inspection of the fruits was carried out starting from t0. Among all the conditions analyzed, in light and cool temperature (12° C) we observed prolonged accumulation of anthocyanins during the three weeks of storage (Fig 1). This was especially visible in the parts of the fruit beneath the sepals, which appeared green at t0, orange/red after the first week, and purple starting from the second week of storage (Fig 1A). Enhancement of anthocyanin accumulation over time was instead not observed when light was absent or at RT (Fig 1B–1D). At RT loss of firmness and fruit spoilage during the third week of storage were clearly visible (Fig 1C and 1D).

**Fig 2:**
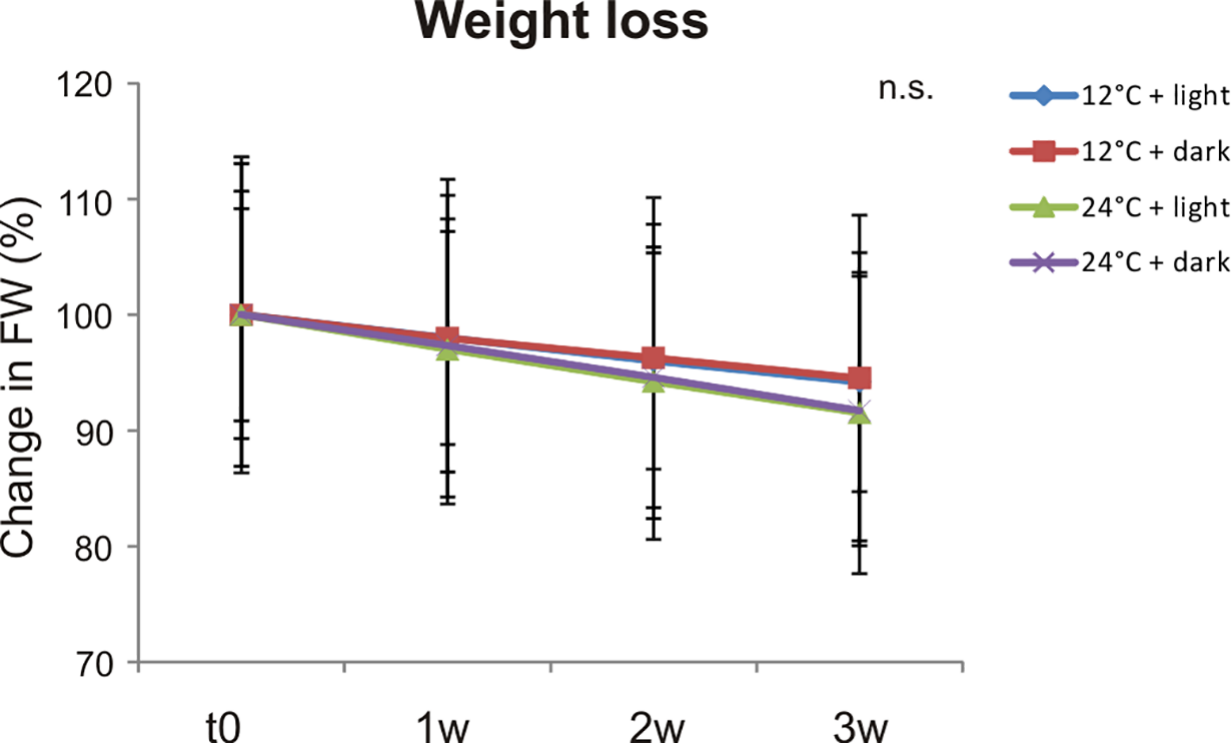
Fruit weight loss during postharverst. Changes in fresh weight (FW) of the *Aft/Aft atv/atv* fruits during the three weeks (w) of storage in the four different conditions (12° C + light, 12° C + dark, 24° C + light and 24°C + dark), expressed as a percentage of weight reduction from the FW measured at the time of collection (t0) ± standard error (n = 5). n.s. stands for “not statistically significant”.

The percentage of fruit weight loss was measured during the three weeks of storage. Even if the differences were not statistically significant, weight loss changes in fruits stored at 12° C appeared on the whole lower than those measured in fruits stored at RT (Fig 2), confirming their higher firmness as visually estimated.

### Analysis of anthocyanin content and other qualitative parameters in fruit stored at 12° C

**Fig 3:**
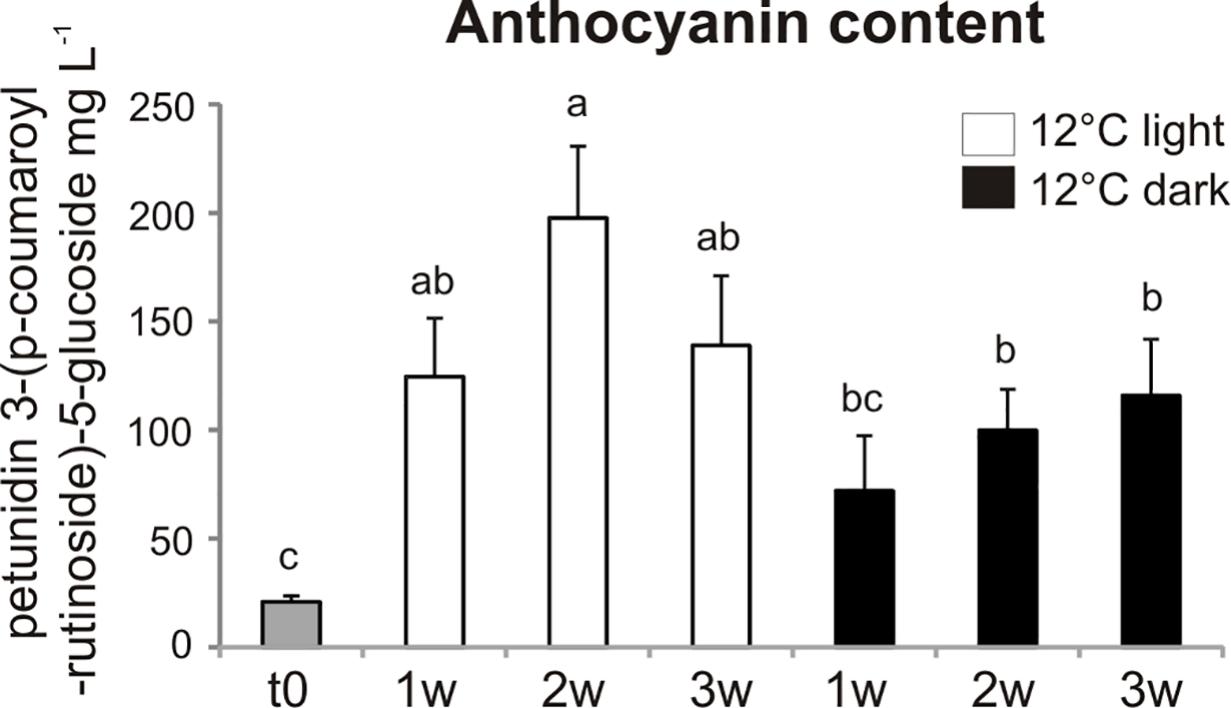
Fruit changes in anthocyanin content during postharvest at 12° C. Anthocyanin content measured in *Aft/Aft atv/atv* fruits at the time of collection (t0 = breaker stage) and during the three weeks (w) of storage in light or dark. The results are means of five replicates ± SE. The values which differ one from another significantly (p < 0.05) are labelled with different letters.

Since the condition of 12° C appeared to better preserve firmness and to increase anthocyanin content of *Aft/Aft atv/atv* fruits after collection, we focused our attention on the group of fruits stored at this temperature. A spectrophotometric analysis to quantify monomeric anthocyanins was carried out on the liquid extracts obtained from whole fruits (skin and flesh) collected at t0 and during the three weeks of storage. Anthocyanin content tended to increase in fruits stored either under light or dark when compared to t0, and the highest accumulation, particularly significant at the second week of storage, was measured in the fruits kept in the presence of light (Fig 3).

**Fig 4:**
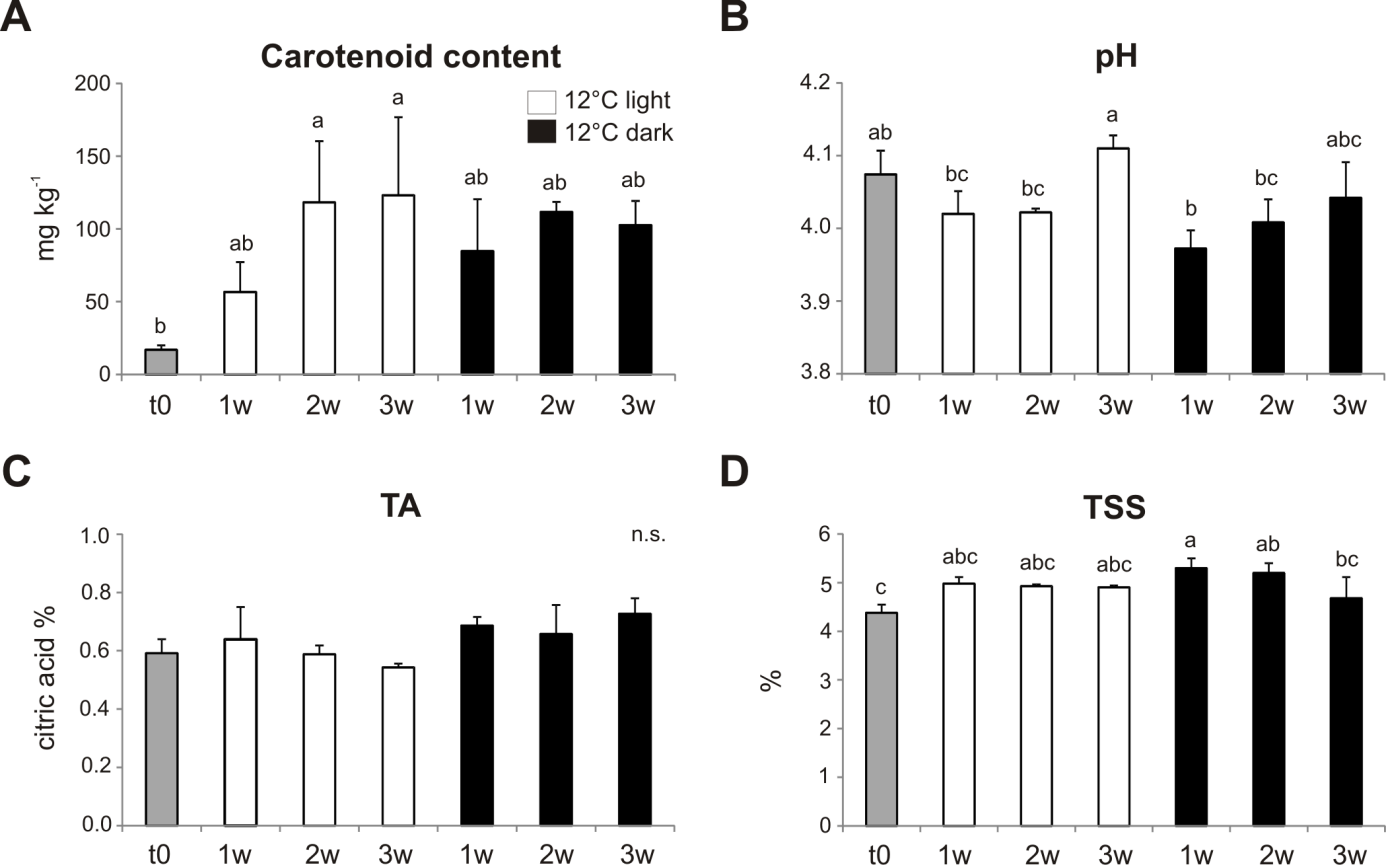
Analysis of the major qualitative parameters in fruits stored at 12° C. Carotenoid content (A), pH (B), titratable acidity (TA) (C), and total soluble solids (TSS) (D) in the whole *Aft/Aft atv/atv* fruits at the time of collection (t0) and after one, two and three weeks (w) of storage under light or in darkness. The results are means of five replicates ± SE. The values which differ one from another significantly (p < 0.05) are labelled with different letters. n.s. stands for “not statistically significant”.

Carotenoid content increased during the three weeks of storage compared to t0, as expected during normal ripening of tomato fruits, but no differences could be observed between light and dark conditions (Fig 4A). pH, titratable acidity and total soluble solids resulted also very similar in fruits stored under light or in the dark and did not significantly differ from t0 (Fig 4B–4D).

### Expression profile of genes involved in anthocyanin and hormones biosynthetic pathways during storage

**Fig 5:**
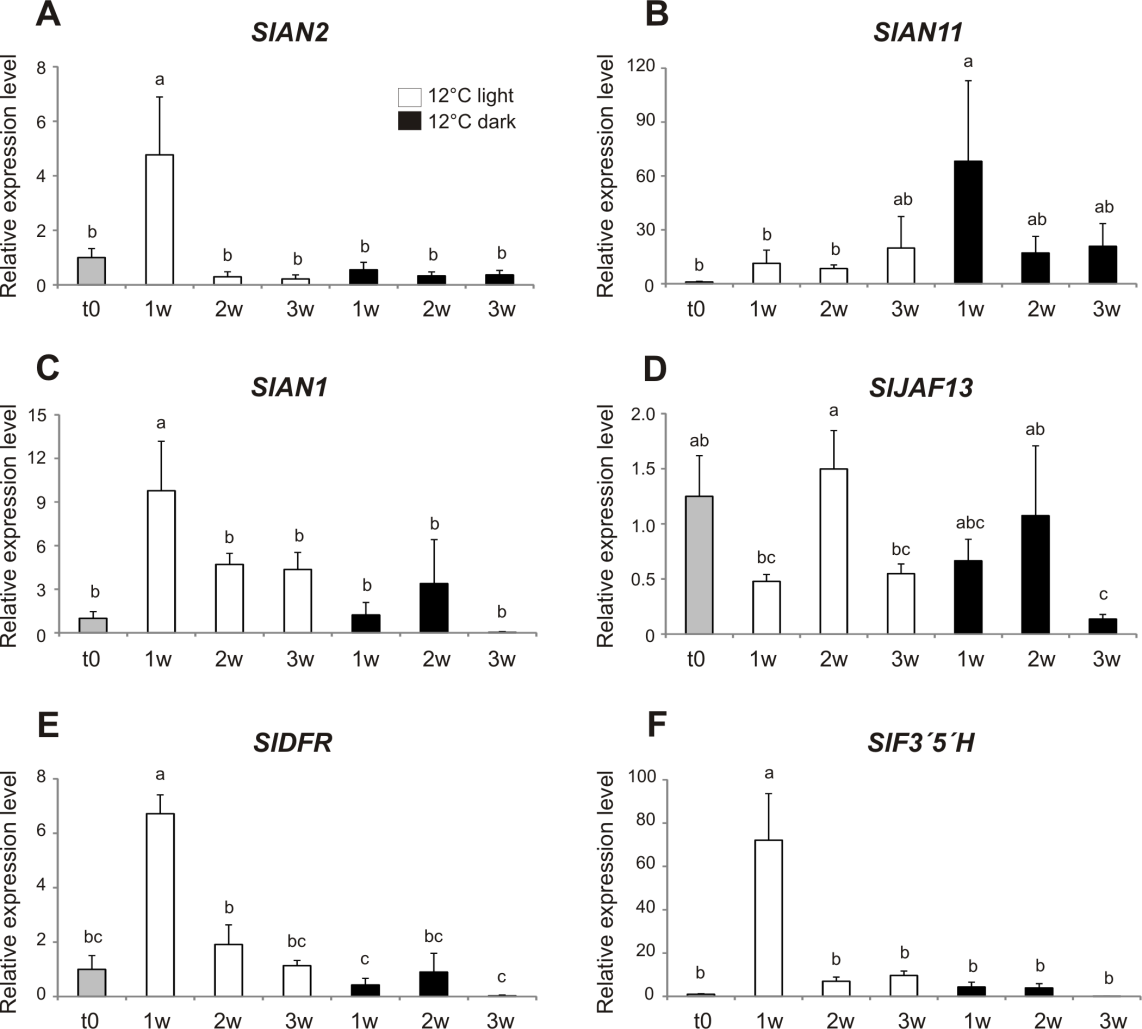
Transcriptomic analysis of genes involved in anthocyanin synthesis in fruits during postharvest at 12° C. qPCR analysis of some regulatory (*SlAN2*, *SlAN1*, *SlJAF13*, *SlAN11*) and structural (*SlDFR*, *SlF3′5′H*) genes possibly involved in the activation of the anthocyanin pathway in the fruit peel of *Aft/Aft atv/atv* fruits. Expression levels were measured in fruits collected at breaker (t0) and stored for one, two and three weeks (w) at 12° C under light or dark. Expression levels are shown as relative units with the value of one of the biological replicates of t0 fruits set to one. Data are means of three biological replicates ± SE. The values which differ one from another significantly (p < 0.05) are labeled with different letters.

We investigated the expression of genes involved in the anthocyanin biosynthetic pathway in *Aft/Aft atv/atv* tomato fruits during storage at 12° C. Both regulatory and structural genes [1] were analyzed. Among the regulatory genes, *SlAN2*, encoding a key R2R3 MYB factor (Fig 5A), and *SlAN1*, encoding a bHLH factor (Fig 5C), were rapidly induced after fruit harvest (t0) in the presence of light. A similar trend was shown by the late structural genes of the anthocyanin biosynthetic pathway, namely *SlF3′5′H* and *SlDFR*, both of which appeared to be induced (compared to t0) after one week of storage at 12° C under light (Fig 5E and 5F). The expression of the other regulatory genes analyzed, *SlJAF13* and *SlAN11*, encoding another bHLH factor and a WD40 protein, respectively, was instead more variable and apparently not influenced by either storage in the light or darkness (Fig 5B and 5D). The R2R3 MYB encoding gene *SlANT1*, another important anthocyanin regulatory gene in tomato [1], was expressed at barely detectable levels (average Ct = 35), without showing differences among the samples (S1 Fig).

The expression analysis of some genes involved in the production of hormones related to fruit ripening and senescence was finally carried out to check if these processes could have been affected by the presence or absence of light during postharvest at 12° C. Abscisic acid (ABA) production was estimated following the expression level of *SlNCED1*, a key gene involved in its biosynthesis in tomato fruits during ripening [40]. The expression of this gene was found in all the samples and decreased over time in a very similar way in light or dark (S2A Fig). Ethylene production, on the other hand, was followed by analyzing the expression of three different genes, *SlACS2*, *SlACS4* and *SlACO1*, all involved in its biosynthesis during fruit ripening [41]. These three genes appeared to be expressed in all the fruits analyzed and showed a higher induction in dark compared to light during the first week of storage (S2B–S2D Fig).

## Discussion

The establishment of protocols that can extend the shelf life of horticultural produce without affecting their quality traits is of great economical importance, given the scale of fruit and vegetable losses worldwide, mostly occurring in postharvest [42]. Many efforts have been undertaken to understand and improve storage conditions of many fruits and vegetables, including tomatoes [43–47]. However, only a few studies were performed dealing with this aspect in anthocyanin-rich tomatoes. Studies were performed using high-anthocyanin transgenic tomatoes [4, 11] or naturally derived anthocyanin-rich fruits but focusing on single storage parameters [10, 23].

Our goal was to establish optimal conditions for the postharvest storage of the *Aft/Aft atv/atv* anthocyanin-rich tomato fruits produced by conventional breeding, where anthocyanins are accumulated on the peel. We did not focus on standard treatments used to prolong shelf life of tomato fruits, since anthocyanin-rich tomatoes are normally characterized by a longer shelf life [4, 11, 23], but took mainly into consideration those peculiar environmental factors which can also affect anthocyanin synthesis or accumulation, namely light and temperature [28, 48]. Therefore, we exposed *Aft/Aft atv/atv* fruits, collected at the breaker stage, to different temperature and light regimes to test if their naturally long shel life can be exploited to maintain or even increase the content of anthocyanins. Our results indicate that exposure at a combination of both moderately low temperatures (12° C) and light represents a suitable storage condition, able to enhance the anthocyanin content (Fig 3) without negatively affecting other quality-related traits (Fig 4).

The choice of the moderate low temperature treatment was due to the fact that tomatoes are often stored at temperatures close to 12° C after harvest and during the pre-marketing period [4, 49], and previous studies showed that cool temperatures (10°-13° C) can be beneficial to prolong tomato shelf life without compromising flavor and taste 43–46]. Moreover, cold represents an important environmental signal for the production of anthocyanins, acting mainly on the transcription of the genes encoding the bHLH components of the MBW complex starting the anthocyanin biosynthetic pathway in plants [26, 32].

Postharvest studies on tomato fruits are mainly carried out in the dark [50]. However, light plays a very important role in fruit pigmentation, as exemplified by the *hp* mutants of tomato, which show in their fruits an increased content of phytonutrients, mainly lycopene and other carotenoids, associated with an enhanced light signaling machinery [51]. On the other hand, light represents a developmental signal necessary for the transcriptional activation of the R2R3 MYB TFs, such as tomato SlAN2, which take part to the MBW complex inducing anthocyanin production [1]. Actually, low temperature together with light exposure resulted effective in increasing anthocyanin accumulation in detached *Aft/Aft atv/atv* fruits (Fig 3), which is in agreement with a positive effect of light and/or low temperatures in fruits still attached to the plant [21].

Many studies indicate that short or pulsed red or UV light treatments on tomatoes during postharvest can be beneficial for their physical-chemical properties and antioxidant compounds, mainly increasing the carotenoids content [52, 53]. Recently, also light emitting diode (LED) systems were evaluated as an efficient artificial lighting technique in tomato postharvest for increasing the commercial and organoleptic quality parameters, particularly firmness, titratable acidity and lycopene concentration [50]. It would be therefore very interesting to extend this kind of studies to anthocyanin-enriched tomatoes, to ascertain if the positive effects exerted on their peel pigmentation by continuous light exposure can be confirmed or even enhanced by the implementation of pulsed or LED light treatments.

The carotenoid content, pH, titratable acidity and total soluble solids in *Aft/Aft atv/atv* fruits stored at 12° C did not differ from those commonly measured at ripening in normal tomato varieties without anthocyanins. A pH in the range of 4.0–4.5 and a TSS in the range of 3.5–5.5% can be considered satisfactory in fresh tomatoes [54]. Also the acidity level and the final amounts of carotenoids accumulated, mostly of which are represented by lycopene [19], are in the standard range. In particular, carotenoids, whose biosynthesis can be negatively affected by low storage temperatures [49, 54], reached final levels which are comparable with those of many commercial tomato varieties [54, 55]. Cherry tomatoes are usually rich in carotenoids [55] and this can explain the high values we measured in our *Aft/Aft atv/atv* fruits, belonging to a cherry variety. The presence of anthocyanins in the peel of *Aft/Aft atv/atv* tomato and their prolonged synthesis during postharvest did not seem therefore to negatively interfere with the main qualitative properties of this kind of fruit (Fig 4).

A qPCR analysis was performed to study changes in the expression of the genes involved in the anthocyanin biosynthetic pathway during storage. These genes can be divided into regulatory and structural ones, the first encoding proteins involved in the multiprotein MBW complex activating the transcription of the second gene group [25]. Among the regulatory genes, a primary role is carried out by those encoding the R2R3 MYB, the bHLH and the WD40 proteins participating in the MBW complex [25, 56]. Most of the genes already known to be involved in the anthocyanin synthesis in tomato [1, 26, 27] appeared to be expressed in *Aft/Aft atv/atv* fruits at the time of collection and also during storage (Fig 5). Only after three weeks of storage in the dark, we measured a general reduction of the mRNA level in tomato peel, attested by the low level of expression of many genes analyzed (Fig 5). Among the regulatory genes, both the R2R3 MYB gene *SlAN2* and the bHLH-encoding one *SlAN1*, whose proteins are putative members of tomato MBW complex [1, 26], were clearly expressed in all the conditions tested and showed a significantly higher expression at 12° C plus light after one week of storage (Fig 5A and 5C). The other putative components of the MBW complex, SlAN11 [27] (Fig 5B), and SlJAF13 [1] (Fig 5D), appeared to be present in all the conditions tested. Coherently with the expression of the regulatory genes, the structural genes analyzed showed the highest expression level after one week of storage at 12° C under light (Fig 5E and 5F). Remarkably, the R2R3 MYB regulatory factor SlANT1, which represents one of the other positive activators of the anthocyanin pathway in tomato plants [1, 57], seemed to be not involved in the pigmentation of the peel during these stages of fruit ripening, being the expression levels of its encoding gene barely detectable and without changes during storage (S1 Fig). On the whole, qPCR data confirmed the biochemical analyses, indicating that the combination of light and moderately low temperature during storage can be particularly favourable for *Aft/Aft atv/atv* fruits, allowing them to prolong the production of anthocyanins already started on the plant by “reactivating” the biosynthetic pathway at the gene expression level, and thus to increase their final amount, which is directly linked with the fruit nutritional value.

A previous study on the physiology of ripening of *Aft/Aft atv/atv* tomatoes revealed modified hormone equilibrium with respect to non-anthocyanin rich fruits during the different ripening stages [23]. Ethylene production, in particular, showed its typical climacteric peak shifted from turning point to mature red in *Aft/Aft atv/atv* tomatoes. This delay correlated well with the higher firmness shown by the *Aft/Aft atv/atv* fruits compared with the wild type ones and was related to their anthocyanin accumulation [23]. Our analysis on the gene *SlNCED1*, involved in ABA biosynthesis [40], showed a drop in the expression after the breaker stage (t0) (S2A Fig), confirming what was already known about the highest activation of this gene at this ripening stage, followed by the peak of ABA during the subsequent turning point [41]. It also indicated that ABA production was not affected in postharvest by the presence of light or dark (S2A Fig). In the *Aft/Aft atv/atv* fruits of our study ethylene synthesis was active since the breaker stage (t0), as indicated by the high expression levels of the biosynthetic genes *SlACS2*, *SlAC4* and *SlACO1* [41]. Ethylene production likely increased during postharvest, due to the further induction of the genes (S2B–S2D Fig). However, storage in darkness seemed to enhance the ethylene biosynthetic pathway even more than in light, with fruits stored in darkness showing significantly higher expression levels of the genes during the first week of storage (S2B–S2D Fig). The possible difference in ethylene production in fruits maintained under continuous light could be a consequence of the higher reactivation of the anthocyanin biosynthetic pathway in their peel (Fig 5), in line with the previous study showing an interference of the anthocyanin accumulation with ethylene evolution in tomato fruits [23]. These aspects of the *Aft/Aft atv/atv* fruits’ ripening and postharvest physiology are worthy of further studies.

In conclusion, this study demonstrates that storing naturally derived anthocyanin-rich tomatoes at moderate low temperature, especially in the presence of light, results in enhanced anthocyanin accumulation in their peel, with unchanged flavour-related features up to three weeks after harvesting. This is due to a *de-novo* synthesis of the pigments which further enriches the nutritional value of the fruits, contributing to extend their shelf life, which is positively correlated with the anthocyanin content, as clearly shown in previous studies [10, 11].

## Supporting information

S1 Table Primer sequences used for qPCR. (DOCX)

S1 Fig Transcriptomic analysis of *SlANT1* in fruits during postharvest at 12° C. qPCR analysis of *SlANT1* gene, possibly involved in the activation of the anthocyanin pathway in the fruit peel of *Aft/Aft atv/atv* fruits. Expression levels were measured in fruits collected at breaker (t0) and stored for one, two and three weeks (w) under light or dark. Expression levels are shown as relative units with the value of one of the biological replicates of t0 fruit set to one. Data are means of three biological replicates ± SE. The values which differ one from another significantly (p < 0.05) are labeled with different letters. n.s. stands for “not statistically significant”.
(TIF)

S2 Fig Transcriptomic analysis of genes involved in ABA and ethylene synthesis in fruits during postharvest at 12° C. qPCR analysis of: (A) *SlNCED1*, a gene involved in the production of ABA; and (B-D) *SlACS2*, *SlACS4*, *SlACO1* genes, involved in the biosynthesis of ethylene. Expression levels were measured in fruits collected at breaker (t0) and stored for one, two and three weeks (w) under light or dark. Expression levels are shown as relative units with the value of one of the biological replicates of t0 fruits set to one. Data are means of three biological replicates ± SE. The values which differ one from another significantly (p < 0.05) are labeled with different letters. n.s. stands for “not statistically significant”.
(TIF)
