# Supplementary material for: Optimizing shelf life conditions for anthocyanin-rich tomatoes
Source: PLoS One. 2018 Oct 11;13(10):e0205650. doi: 10.1371/journal.pone.0205650 (PMC6181405; doi:10.1371/journal.pone.0205650)
Supplement: S1 Table — (DOCX) [file pone.0205650.s001.docx]

**S1 Table.** **Primer sequences used for qPCR**

| **Target gene** | **Forward primer (5′-3′)** | **Reverse primer (5′-3′)** |
| --- | --- | --- |
| *SlEF1A^1^* | GCTGCTGTAACAAGATGGATGC | GGGGATTTTGTCAGGGTTGTAA |
| *SlAN2^1^* | TTCCAGGAAGGACAGCAAAC | AACGAGGACGAGAATGAGGA |
| *SlANT1^1^* | ATAAGTCATGAAAATTGGGGTGAAT | AGATTCCATAAGTCAATTTCAGCAG |
| *SlAN1^1^* | CCTCTCTTGGACGGTGTTGT | GCTTGTTGTGGCTCATTGAA |
| *SlJAF13^1^* | TCAGGGGATCACTACCGAAC | TCCCATCAAGGTTGGAAGAC |
| *SlAN11^1^* | ATGAAGTGGAGCCGAGAAGA | TCCATCAGCAGAAACAGA |
| *SlDFR^1^* | TCCGAAGACGACAACGGTTT | TGACAAGCCAAGAGCCGATAA |
| *SlF3′5′H^2^* | GGCAATTGGACGAGATCCTG | AAGGAACCTCTCGGGAGTGAA |
| *SlNCED1^3^* | AGGCAACAGTGAAACTTCCATCAAG | TCCATTAAAGAGGATATTACCGGGGAC |
| *SlACS2^3^* | CTACGCAGCCACTGTCTTTGAC | TGATTCCGACTCTAAATCCTGGTAA |
| *SlACS4^3^* | TTGCGACGAAATATATGCTGCT | CACTCGAAATCCTGGAAAACCT |
| *SlACO1^3^* | ACTATCCACCATGTCCTAAGCCCG | TCTGTTTGTGCAATTACTCTGTGCAGC |

^1^ Kiferle C, Fantini E, Bassolino L, Povero G, Spelt C, Buti S, et al. Tomato R2R3-MYB proteins SlANT1 and SlAN2: Same protein activity, different roles. PLoS ONE. 2015; Aug 26; 10(8):e0136365. doi: 10.1371/journal.pone.0136365.

^2^ Bovy A, de Vos R, Kemper M, Schijlen E, Almenar Pertejo M, Muir S, et al. High-flavonol tomatoes resulting from the heterologous expression of the maize transcription factor genes LC and C1. Plant Cell. 2002; 14: 2509-2526.

^3^ Ji K, Kai W, Zhao B, Sun Y, Yuan B, Dai S, et al. *SlNCED1* and *SlCYP707A2*: key genes involved in ABA metabolism during tomato fruit ripening. J Exp Bot. 2014 ; 65: 5243-5255. doi: 10.1093/jxb/eru288.
