# Supplementary figures and images for: Optimizing shelf life conditions for anthocyanin-rich tomatoes
Source: PLoS One. 2018 Oct 11;13(10):e0205650. doi: 10.1371/journal.pone.0205650 (PMC6181405; doi:10.1371/journal.pone.0205650)

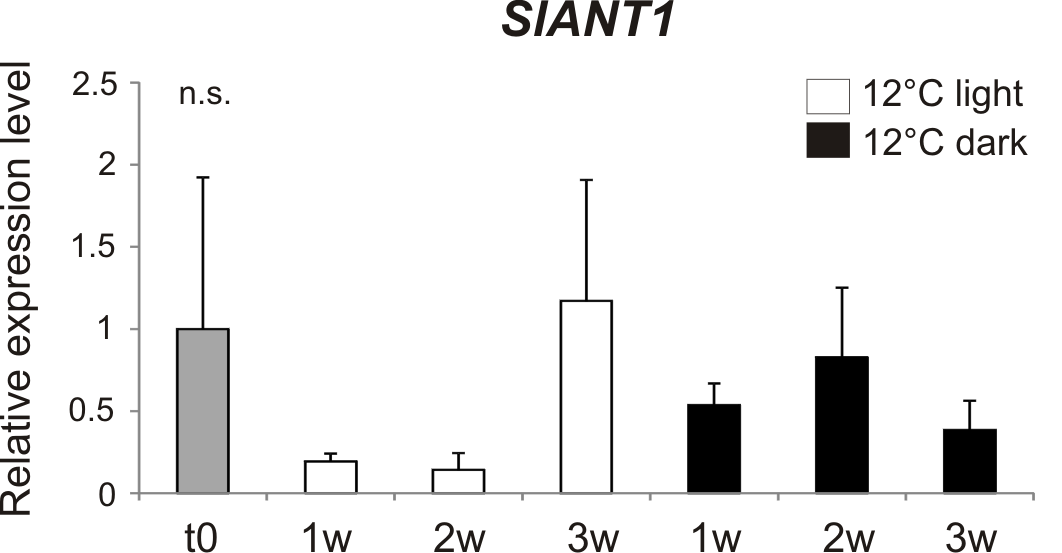

Supplement: S1 Fig — qPCR analysis of SlANT1 gene, possibly involved in the activation of the anthocyanin pathway in the fruit peel of Aft/Aft atv/atv fruits. Expression levels were measured in fruits collected at breaker (t0) and stored for one, two and three weeks (w) under light or dark. Expression levels are shown as relative units with the value of one of the biological replicates of t0 fruit set to one. Data are means of three biological replicates ± SE. The values which differ one from another significantly (p < 0.05) are labeled with different letters. n.s. stands for “not statistically significant”. (TIF) [file pone.0205650.s002.tif]

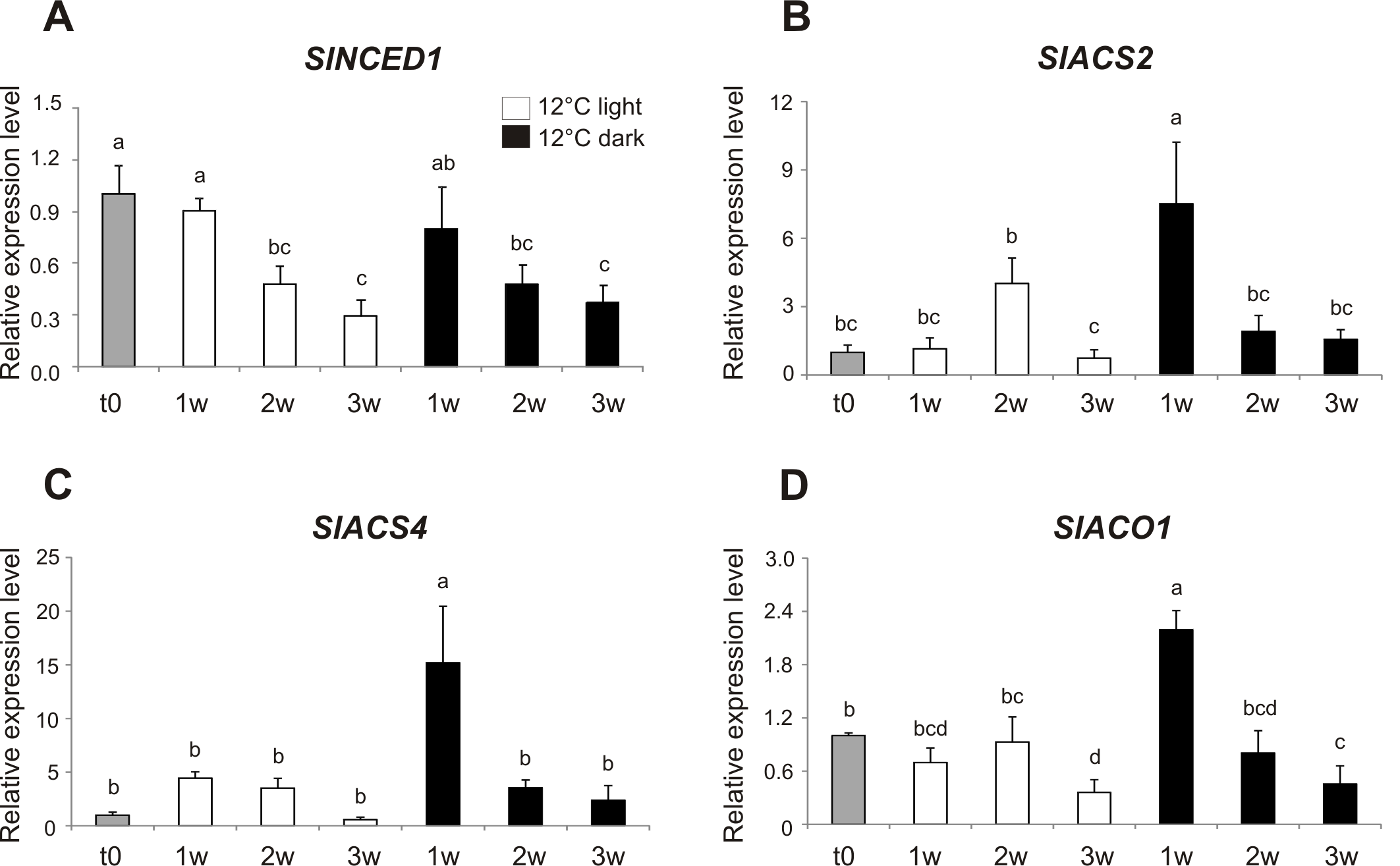

Supplement: S2 Fig — qPCR analysis of: (A) SlNCED1, a gene involved in the production of ABA; and (B-D) SlACS2, SlACS4, SlACO1 genes, involved in the biosynthesis of ethylene. Expression levels were measured in fruits collected at breaker (t0) and stored for one, two and three weeks (w) under light or dark. Expression levels are shown as relative units with the value of one of the biological replicates of t0 fruits set to one. Data are means of three biological replicates ± SE. The values which differ one from another significantly (p < 0.05) are labeled with different letters. n.s. stands for “not statistically significant”. (TIF) [file pone.0205650.s003.tif]
